# Ultrasound with mineral water or aqua gel to reduce pain and improve the WOMAC of knee osteoarthritis

**DOI:** 10.4155/fsoa-2016-0003

**Published:** 2016-02-26

**Authors:** Sahar Ahmed Abdalbary

**Affiliations:** 1 Department of Orthopedic Physical Therapy, MTI University, 2, Street 107, Maadi, Postal Code 11431 Cairo, Egypt

**Keywords:** knee osteoarthritis, mineral water, ultrasound, VAS, WOMAC

## Abstract

**Aims:**

Osteoarthritis is the most degenerative joint disease. The aim was to investigate the effects of ultrasound using mineral water or aqua sonic gel on severity of knee pain, measured by the visual analog scale and the Western Ontario and McMaster Universities Arthritis Index (WOMAC).

**Materials and methods:**

Thirty women with bilateral osteoarthritis of the knee were assigned to two groups: ultrasound with mineral water (group 1, n = 15) or with aqua sonic gel (group 2, n = 15). Both groups underwent 4 weeks intervention, three per week. The participants were assessed using the visual analog scale and the WOMAC. Tests were performed before and after interventions.

**Results:**

Both groups had significantly reduced pain and improved WOMAC compared with preintervention values.

**Discussion:**

The ultrasound with mineral water group had more pronounced improvement at p-value < 0.001.

**Conclusion:**

Ultrasound with mineral water is preferable in treatment of knee OA.

## Lay abstract

Knee pain is terrible for the patient and affects the locomotor activity of the person. Our study compares an ultrasonic device with traditional gel for the treatment of knee pain versus an ultrasonic device with mineral water from natural source. We compared treatment results after 1-month treatment period on the pain and the physical function of these patients. Our results demonstrated that treatment with ultrasound with mineral water showed superior patient improvement in terms of pain and physical function.

Osteoarthritis (OA) is one of the most common chronic and degenerative joint diseases [1]. The joints most affected are the knee, hip, hand, spine and foot joints [2]. OA of the knee is the most common type, with an estimated incidence between 12 and 35% in the general population [3].

OA is a disease of multiple dimensions characterized by deterioration of the articular surface of the joint. OA presents with pain, deformity, muscle atrophy, progressive loss of independence and decrease in functional capability [4]. Risk factors for OA can be divided into person-level factors, such as age, sex, obesity, genetics, race and diet and joint level factors including injury, malalignment and abnormal loading of the joint [5].

Appropriate treatment modalities for all individuals with knee OA included biomechanical interventions, intra-articular corticosteroids, exercise (land based and water based), self management and education. For specific clinical subphenotypes recommended treatments were balneotherapy and pharmacological drugs but, uncertain appropriateness for specific clinical subphenotypes acupuncture and ultrasound [6]

Therapeutic modalities in treating OA are mainly directed at symptom relief and other treatment options, including both nonpharmacological and pharmacological measures. Physiotherapy is one of the recommended nonpharmacological options in patients with OA [7].

One of the most common physical agents used within physiotherapy practice, ultrasound therapy is based on the application of high-frequency sound waves to body tissues in order to obtain mechanical or thermal effects. These effects are intended to enhance soft tissue healing, increase blood flow and metabolic activity and decrease inflammatory response and decrease pain [3].

Ultrasound treatment modalities have been proven to promote repair of full-thickness articular cartilage defects, have resulted in formation of hyaline cartilage life repair tissue at the sites of defects, softened and dissipated condensed fibrous connective tissue and delayed progression of early OA [8].

Ultrasound may be administered in either continuous or pulsed mode. Pulsed ultrasound produces nonthermal effects and is used to aid in the reduction of inflammation, whereas continuous ultrasound generates thermal effects to alleviate pain [9].

When ultrasound is used with specific medicaments to enhance transdermal absorption of a compound, significant amounts of the compound are absorbed by the subcutaneous circulation [10].

Balneotherapy is defined as the use of baths (in tubs or pools) containing thermal and/or mineral water from natural springs or drilled wells [11]. The main mode of balneotherapy includes immersion in thermal water with a natural temperature of at least 20ºC and/or mineral water with total mineral content of at least 1g/l. Spa therapy, which includes balneotherapy, additionally employs other balneological interventions such as mud applications, mineral water drinking, showers, underwater pressure jets or thermal mineral waters and exercise in thermal water pools. Other nonpharmacological therapies (massage, exercise etc.) can be combined with these in spa therapy programs [12].

Spa therapy is one of the most commonly used nonpharmacological approaches for OA in many European and Middle Eastern countries. It comprises a broad spectrum of therapeutic modalities including hydrotherapy, balneotherapy, mud-pack therapy, physiotherapy and exercise [13].

For patients with knee osteoarthritis a short-term 3 weeks of spa therapy with home exercises and usual pharmacological treatments offers benefit after 6 months compared with exercises and usual treatment alone [14].

Mineral water has been used for a long time in the alleviation of musculoskeletal pain. The beneficial effects of balneotherapy were studied and proven in a variety of rheumatological diseases in past decades. In osteoarthritis of the knee, pain and musculoskeletal dysfunction improved in the short to medium terms [15].

In Israel, increased serum concentrations of bromine, rubidium, calcium and zinc were noted in patients with psoriasis after bathing in the Dead Sea. Whether these chemicals have any biologic effect is not known. There are data, however, that indicate that sulfur can be absorbed through the skin and may have analgesic effects [16].

Beneficial effects of a cycle of sulphate–bicarbonate–calcium mineral water baths have been shown for the pain management, functional capacity and quality of the life parameters in patients with knee OA [17].

The aim of this study was to investigate the effects of 12 sessions of pulsed ultrasound with mineral water versus that with aqua sonic gel on severity of knee pain and evaluation of perceived health and physical function using the Western Ontario and McMaster Universities Osteoarthritis Index (WOMAC) in women with bilateral osteoarthritis of the knee.

We hypothesized that there will be greater improvement in severity of knee pain and perceived physical function in the group treated with pulsed ultrasound and natural mineral water than in that treated with pulsed ultrasound and aqua sonic gel.

The procedures followed were in accordance with the ethical standards of the committee on human experimentation of Cairo University. The trial was registered by number: NTR 334.

## Materials & methods

This was a controlled randomized single-blinded assessor study. The block randomization list was kept by individuals who had no contact with the investigator, who assigned patients to their randomization treatment.

Patients with osteoarthritis of the knee were recruited from an orthopedic clinic, evaluated and informed about the objective and experimental procedures of the study. Thirty women with bilateral OA of the knee completed all the procedures. Exclusion criteria included any rheumatic disease (with the exception of bilateral OA of the knee), unilateral OA of the knee, neurological disorders, cognitive limitations or endocrine disease. These also included patients who had undergone arthroscopy or treatment with intra-articular hyaluronic acid during previous 6 months or who had been treated with intra-articular corticosteroids during the past 3 months. Inclusion criteria included female sex, bilateral OA of the knee, Kellgren–Lawrence scores of 2 and 3 on radiologic evaluation [17], age range of 47–67 years, absence of any lower limb disease except bilateral OA of the knee, absence of current physical therapy treatments for OA of the knee and diagnosis of bilateral OA of the knee according to American College of Rheumatology criteria [18].

Participants were randomized into two groups: group 1 received ultrasonic treatments with mineral water (n = 15) and group 2 received ultrasonic treatments with aqua sonic gel (n = 15). These physiotherapy treatments were performed three-times per week for 4 weeks. The patients' characteristics are presented in Table 1.

The study was conducted at a clinic in the spa center. Design was organized into four steps: medical and physical examination; preintervention evaluations; treatment; postintervention evaluations. Participants underwent detailed medical examination (performed by an orthopedist) and OA diagnostic evaluation (dependent on symptoms and weight-bearing antero–posterior and lateral knee radiographs). One day before and after treatment, participants were evaluated for perceived health and physical function using the WOMAC questionnaire [19,20], and for pain using the visual analog scale (VAS) [21]. Patients underwent 4 weeks of treatment, three-times per week. The entire treatment program was approved by the University Human Research Committee. All patients signed informed consent forms before participating in the study. For both groups we advised them to discontinue established pharmacological treatments such as acetaminophen or NSAIDs before 1 week of preintervention assessment and till the postintervention assessment.

## Treatment program

All patients completed 4 weeks of treatment (12 sessions). The ultrasonic protocol for each group consisted of pulsed ultrasonic waves of 1 MHz frequency and 1 W/cm^2^ power, applied with a 5-cm diameter applicator (Sonopuls 492, 1–3 MHz, Enraf-Nonius, The Netherlands). The area of treatment was cleaned to permit an effective skin applicator coupling. For group 1 we used the indirect ultrasound applications, degassed water because it is less expensive [22]. Group 1 patients were each placed in a supine position and mineral water (composition detailed in Table 2) was applied. Ultrasound was then applied to the medial and lateral aspects of the knee in circular motions, with the probe at right angles to ensure maximum absorption of energy. Group 2 patients received an application of aqua sonic gel, free of any pharmacologic active ingredients, along with the ultrasound, in the same manner as the Group 1 patients. Each session lasted 5 min. The patients were assessed for severity of pain and perceived health and physical function using the WOMAC before and after treatment.

## Severity of knee pain

Knee pain was assessed using the VAS. The VAS consists of a 10-cm line, with the extreme left indicating ‘no pain’ or ‘0’ and the extreme right indicating ‘unbearable pain’ or ‘10’. The patients were instructed to use the scale to indicate their current level of pain. The values (in centimeters) were recorded for the statistical analysis.

## WOMAC osteoarthritis index

The WOMAC osteoarthritis index was used as a subjective measure of perceived health and physical function.

The WOMAC osteoarthritis index is a three-part questionnaire, completed by the patients themselves and consisting of 24 questions revealing clinically important symptoms in the areas of pain (5 questions), stiffness (2 questions) and physical function (17 questions) for patients with OA of the hip and/or the knee [14,15]. In the present study, we used a Likert scale version of the WOMAC that allows patients to state their responses using a five-point scale (0 = none, 1 = mild, 2 = moderate, 3 = severe, 4 = extreme). The higher the score achieved, the lower the level of perceived health and physical function. Scores were generated for the three dimensions of pain, stiffness and physical function by summing the coded responses. The patients were instructed to answer all the questions.

The WOMAC questionnaire has been proven to show moderate to high validity in many studies [23].

## Statistical analysis

Power analysis (Θ = 0.05, X = 0.80) determined that a sample size of 15 patients in each group was needed to demonstrate a 10-mm difference in improvement in the patient's assessment of pain (using 0–100-mm VAS) between the both groups, with standard deviation of 22 mm.

The data were analyzed using the Statistical Package for the Social Sciences version 16 software for windows (SPSS Inc, IL, USA). Data were represented as mean and ± SD. All variables presented normal distributions according to Kolmogorov–Smirnov tests. Paired t-sample t-tests were used to compare pre- and post-treatment changes in each group. Student's T-tests were used to compare both groups. The results were considered to be statistically significant at p < 0.05.

## Results

All participants completed 12 treatment sessions (Table 1) representative of the demographic data and clinical characteristics of both groups. Both groups did not differ significantly with respect to age, BMI, symptom duration or Kellgren–Lawrence grade.

Table 3 represents data with respect to evaluation of bilateral knee joint pain by VAS (cm), pain by WOMAC, rigidity by WOMAC, physical function by WOMAC and total WOMAC scores of both groups. Before treatment, we found no significant differences between the group treated with ultrasound and mineral water (group 1) and that treated with ultrasound and aqua gel (group 2). However, in the intragroup comparisons (before vs after), a significant decrease was observed in the VAS for pain and the total WOMAC scores. The scores for each dimension in both experimental groups with p < 0.05 are presented in Tables 4 & 5. Compared with the baseline, significant improvements were observed in both groups at the end of treatment. The improvement in the group treated with ultrasound and mineral water (group 1) was more highly pronounced than that of those treated with ultrasound and aqua gel (group 2; p < 0.05) (Table 6).

## Discussion

OA of the knee is considered to be the fourth highest cause of disability in women and is responsible for the deterioration of quality of life and functional ability [24]. Evidence suggests that mineral water is safe and effective for treating patients with OA of the knee, and may be considered as one treatment option among many approaches in treating this disease [11]. Many studies have investigated how different types of non-pharmacological treatments affect pain and WOMAC index scores [1,13,24].

To our knowledge, this is the first study that examines the therapeutic effects of mineral water in combination with ultrasound compared with the traditional method of using aqua gel and ultrasound in the treatment of OA of the knee.

Mineral baths seem to be more beneficial to quality of life when compared with an absence of pain treatments and/or analgesic intake. It was presumed that most mineral ingredients are absorbed through the skin into systemic circulation. This mechanism has not been confirmed so far [25].

In this present study, significant beneficial effects on pain and functional capacity in patients with OA of the knee were demonstrated using mineral water with ultrasound. All evaluation parameters were significantly reduced after the treatment. A significant difference, superior to that of the group in which aqua gel was used with ultrasound, was observed. Our results have shown that using mineral water with ultrasound improves pain and total WOMAC scores.

In this study we proposed that the absorption of the components of mineral water is enhanced by ultrasound, resulting in benefits additional to those of conventional therapeutic ultrasound using aqua gel, however effective both treatment methods are.

Ultrasound is one of the deep heating modalities used in physical therapy. Therapeutic ultrasound is generated by a transducer that converts electrical energy to ultrasound by using the piezoelectric principle [25]. Although the exact mechanism of action is unknown, one of the important healing properties of ultrasound is its ability to increase regional blood flow and connective tissue extensibility. Nonthermal effects may be related to molecular vibration that increases cell membrane permeability and enhances metabolic product transport [26].

In our study, we used mineral water (containing cations and anions) with ultrasound for one group, and ultrasound with aqua gel in another group. Therapeutic ultrasound administration enhances percutaneous penetration of topical diclofenac gel [27]. We propose that deeper absorption of certain components of mineral water results in clinical benefits in addition to those of the ultrasound treatment. Nevertheless, both methods were found to be effective. The exact mechanism of action of mineral water is unknown, but the minerals absorbed from the water may have a therapeutic role [11].

Our results have shown that using mineral water with ultrasound treatment may be useful alongside pharmacological therapy, and may represent an alternative option to patients with OA of the knee and who are susceptible to drug-related side effects.

It is difficult to separately analyze the effect of each component of mineral water. Spa water, which contains high sulfide ions (S^-2^), is dominant. Sulfur, which can be absorbed into the body through the skin, has a known antioxidant effect in the cellular level. The antioxidant effect of sulfurous mineral water protects cartilage tissue against the oxidative damage in osteoarthritis [28–30].

The efficacy is probably the result of a combination of factors covering ultrasound and chemical effects of the mineral water.

We used the pulsed ultrasound in our study because pulsed ultrasound with greater probability of being the preferred mode, is more effective in both pain relief and function improvement, whereas the continuous ultrasound could only be considered as a pain relief treatment in the management of knee OA [31]. In our study, we used the pulsed ultrasound to remove the effect of heat on the mineral water compositions.

Our sample is homogeneous because we recruited only women with bilateral OA of the knee.

## Study limitations

The main limitations of this study are: the small number of patients; the short period of treatment of 1 month and 12 sessions; the absence of follow-up after the treatment and that it did not include any quality of life outcome. Other, more objective outcome variables should be included in the study protocol, such as range of motion at the knee joint, among others.

## Conclusion

Ultrasound treatment is effective for reducing pain and improving WOMAC scores. However, ultrasound treatment with mineral water is superior to ultrasound treatment with aqua gel. The results of the present study may encourage subsequent researchers with larger patient populations and the ability to follow-up to verify therapeutic effects and to assess the biological effects of mineral water with ultrasound on the clinical status of patients with OA of the knee.

## Future perspective

Mineral water and ultrasound may be useful and may represent an alternative treatment in patients with OA with a high risk to drugs side effects. We can extract from the active ingredient composition of the water ointment to be used with the ultrasound and may be also extract drugs to be used in treatment of the knee OA.

**Table 1.** **Demographic and clinical characteristics of patients.**

**Character**	**Group 1 (n = 30)**	**Group 2 (n = 30)**	**p-value**
Age (years)	51.03 ± 6.5	50.63 ± 6.2	0.81
BMI (kg/cm2)	34.34 ± 6.4	33.75 ± 6.5	0.73
Duration of symptoms (years)	5.5 ± 1	5.1 ± 0.9	0.43
Kellgran–Lawrence (grade 2/grade 3)	2.63 ± 0.5	2.53 ± 0.491	0.44

Data are expressed as mean ± SD, with p < 0.05.

**Table 2.** **Mineral compositions of water used in the study.**

**pH**	**7.15**
Conductivity	26.23 ms
Sodium (Na^+^)	2300 ppm
Potassium (K^+^)	163 ppm
Calcium (Ca^+2^)	1002 ppm
Magnesium (Mg^+2^)	753 ppm
Total hardness (CaCO_3_)	5600 ppm
Ammonia (NH_4_)	0.19456 ppm
Chloride (Cl)	7810 ppm
Copper (Cu)	0.01 ppm
Manganese (Mn^2+^)	10 ppb
Chromium (Cr^5+^)	20 ppb
Iron (Fe^2+^)	0.752 ppm
Phosphate (Ph^2+^)	1.5 ppm
Sulfur (S^2-^)	5 ppb
Floride (F^-^)	2 ppm
Sulfate (SO_4_^2-^)	10 ppm
Nitrate (NO_2_)	7.531ppm

**Table 3.** **Comparison between groups before treatment.**

**Variable**	**Group: ultrasound + water**	**Group: ultrasound + gel**	**p-value**
VAS	6.8 ± 1.1	6.6 ± 1	0.476
Pain WOMAC	16.9 ± 1.3	14.97 ± 1.87	0.216
Rigidity WOMAC	6.1 ± 0.81	5.63 ± 0.72	0.789
Physical function WOMAC	43.9 ± 6.64	38.8 ± 8.34	0.391
Total WOMAC	66.9 ± 6.88	60.47 ± 9.18	0.366

VAS: Visual analogue scale; WOMAC: Western Ontario and McMaster Universities osteoarthritis index.

**Table 4.** **Comparisons in Group 1 (ultrasound + water) before and after treatment.**

**Variable**	**Before treatment**	**After treatment**	**p-value**
VAS	6.8 ± 1.1	1.47 ± 0.9	0.001^†^
Pain WOMAC	16.9 ± 1.3	1.4 ± 0.89	0.001^†^
Rigidity WOMAC	6.1 ± 0.81	0.73 ± 0.58	0.001^†^
Physical function WOMAC	43.9 ± 6.64	5.27 ± 2.83	0.001^†^
Total WOMAC	66.9 ± 6.88	7.43 ± 3.1	0.001^†^

^†^Statistically significant.

VAS: Visual analog scale; WOMAC: Western Ontario and Mc Master Universities osteoarthritis index.

**Table 5.** **Comparisons in Group 2 (ultrasound + gel) before and after treatment.**

**Variable**	**Before treatment**	**After treatment**	**p-value**
VAS	6.6 ± 1	2.6 ± 0.86	0.001^†^
Pain WOMAC	14.97 ± 1.87	4.2 ± 1.54	0.001^†^
Rigidity WOMAC	5.63 ± 0.72	2.77 ± 0.77	0.001^†^
Physical function WOMAC	38.8 ± 8.34	20.43 ± 3.18	0.001^†^
Total WOMAC	60.47 ± 9.18	27.33 ± 3.75	0.001^†^

^†^Statistically significant.

VAS: Visual analog scale; WOMAC: Western Ontario and Mc Master Universities osteoarthritis index.

**Table 6.** **Comparison between both groups after treatments.**

**Variable**	**Group ultrasound + water**	**Group ultrasound + gel**	**p-value**
VAS	1.47 ± 0.9	2.6 ± 0.86	0.001^†^
Pain WOMAC	1.4 ± 0.86	4.2 ± 1.54	0.001^†^
Rigidity WOMAC	0.73 ± 0.58	2.77 ± 0.77	0.001^†^
Physical function WOMAC	5.27 ± 2.83	20.43 ± 3.18	0.001^†^
Total WOMAC	7.43 ± 3.07	27.33 ± 3.75	0.001^†^

^†^Statistically significant.

VAS: Visual analog scale; WOMAC: Western Ontario and McMaster Universities osteoarthritis index.

**Executive summary**

Ultrasound treatment with the mineral water was superior in treatment of bilateral knee osteoarthritis in female patients after 12 sessions of treatment.
The superiority was determined at pain measured by visual analog scale.
The superiority was also determined at the Western Ontario and McMaster Universities osteoarthritis index.
